# Ethical Management of Challenging Behaviors in Hospitalized People Who Use Drugs

**DOI:** 10.1093/cid/ciaf231

**Published:** 2025-05-23

**Authors:** Christina Yen, Kinna Thakarar, Tim Lahey

**Affiliations:** Department of Medicine, MaineHealth Maine Medical Center, Portland, Maine, USA; Department of Medicine, Tufts University School of Medicine, Boston, Massachusetts, USA; Department of Medicine, MaineHealth Maine Medical Center, Portland, Maine, USA; Department of Medicine, Tufts University School of Medicine, Boston, Massachusetts, USA; Department of Medicine, University of Vermont Larner College of Medicine, Burlington, Vermont, USA; Department of Medicine, University of Vermont Medical Center, Burlington, Vermont, USA

**Keywords:** ethics, clinical, substance use disorder, hospitals, role conflict

## Abstract

From drug use in the hospital to patient-directed discharges and threatening behavior, challenging behaviors arise frequently in inpatients with infectious complications of substance use disorders (SUDs). The management of such challenging behaviors can bring key ethical values into tension and be susceptible to clinician bias. Here, we characterize the ethical tensions that emerge in the management of challenging behaviors in inpatients with infectious complications of SUD, identify preventive approaches, and delineate how clinicians can respond when preventive measures fail. Such preventive and post hoc responses incorporate the principles of harm reduction as well as of patient-centered care and trauma-informed care.

## Teaser

- Challenging behaviors in inpatients with infectious complications of substance use disorder can bring core ethical values into tension, ranging from respect for patient autonomy to institutional resource allocation.
- Harm reduction–based care, ranging from provision of safer use supplies and addiction consult services to less restrictive institutional policies, can prevent or ameliorate such ethics conflicts and, as a result, should be standard.
- If prevention efforts fail and ethical conflicts arise, categorizing challenging behaviors by disease severity and behavioral risk, reflecting on internal biases, and working collaboratively across disciplines can identify the best resolution to such challenging scenarios.

Hospitalization rates for the infectious complications of substance use disorder (SUD) have exploded in recent years [1, 2]. Compared with other hospitalized patients, hospitalized patients with SUD suffer alarming morbidity and mortality plus longer lengths of stay, worse 30-day readmission rates, and more frequent patient-directed discharge [3–8].

One of the most difficult and potentially divisive aspects of the care of people who use drugs (PWUD) when hospitalized with infections is the management of challenging behaviors. Challenging behaviors—a phrase used to signal difficulty faced by clinical staff without implying that such behaviors are necessarily illogical, blameworthy, or wholly patient-driven—can include drug use or intoxication in the hospital, prolonged departures from the inpatient unit, patient-directed discharges, and threatening or violent behaviors. Challenging behaviors may be associated with any form of SUD. The ethical tensions discussed here are not substance-specific.

After presentation of an illustrative case, we discuss the ethical tensions elicited by the challenging behaviors in hospitalized PWUD, summarize approaches to prevention, and close with recommended team-based responses when prevention fails.

## CASE

Mr. F is hospitalized for group A streptococcal bacteremia from xylazine wounds [9]. The hospital medicine and infectious diseases teams are discussing Mr. F's care.

Previous conversations concerned the necessary duration of intravenous penicillin, xylazine wound care, and in-hospital initiation of medications for opioid use disorder (MOUD). Mr. F missed doses of his intravenous penicillin while he was unexpectedly away from the unit, but feedback improved his availability. This morning, the team is discussing the discovery of drug equipment in Mr. F's hospital room.

Overnight, a nurse working with an intravenous line found a bloody needle in Mr. F's bedclothes. “It could have stabbed me!” the nurse said. When the nurse raised this concern with Mr. F, he became irate. He denied using drugs, said the nurse was uncaring, and, using a string of expletives, declined to have his room or possessions searched.

The bedside nurse and nurse leader believe the team should discharge Mr. F. “He can’t use our hospital like a drug den or abuse us at work. If we don’t enforce our own hospital's rules,” said the unit nursing leader, “it's a free for all!” They refer to the hospital's patient rights and responsibilities policy, which prohibits in-hospital drug use and requires respectful treatment of healthcare workers.

The hospitalist said, “He's still bacteremic. He could die if we discharge him!”

How should the team proceed?

## ETHICAL TENSIONS

**Figure 1.**
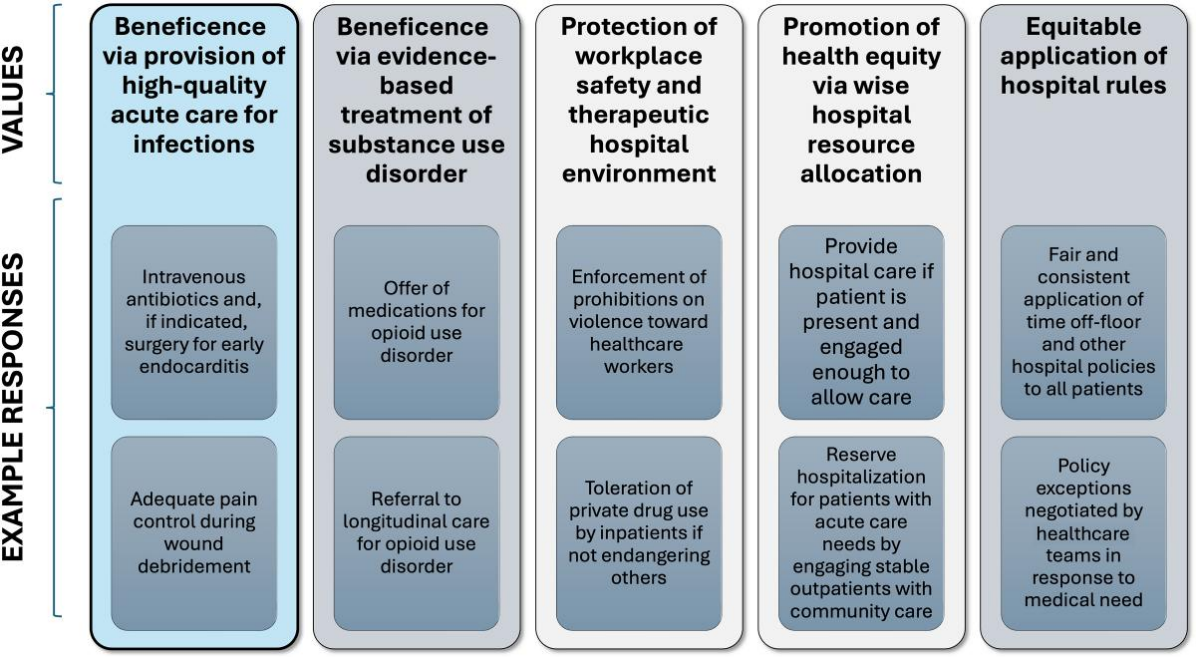
Ethical values involved in behavioral management of hospitalized people who use drugs.

Multiple principles of bioethics relate to the care of PWUD, including respect for patient autonomy, beneficence, nonmaleficence, protection of workplace safety, promotion of health equity, and wise institutional resource allocation, as depicted in Figure 1.

Tensions between these ethical values can arise in the hospital care of PWUD. When a patient with endocarditis after injection drug use threatens healthcare workers, prescribers may prioritize one value, that is, beneficence in the form of antibiotic treatment and surgical care, whereas nurses, who are more exposed to violence in hospitals [10], may advocate for behavior control measures meant to protect workplace safety.

When managing challenging behaviors in hospitalized PWUD, healthcare teams should develop patient-specific solutions that balance ethical values in a fashion appropriate for the severity of the patient's disease, the magnitude of risks associated with their behavior, and the patient’s response to preventive interventions while avoiding undue influence from stigma.

In each patient situation, clinical teams must identify which ethical value is preeminent at the time. Ensuring workplace safety is the preeminent value when a stable patient with an injection site abscess is violent toward healthcare workers. By contrast, when an unstable patient is found to have drug equipment in their hospital room, restoration of patient stability as required by the value of beneficence supersedes other priorities, such as institutional workplace safety–centered prohibitions of patient substance use.

Correct identification of preeminent and subsidiary ethical values and an individualized way to harmonize them require healthcare team collaboration. Team collaboration can ameliorate conflict, mitigate clinician bias, and ensure inclusion of key stakeholder concerns.

Challenging behaviors in hospitalized PWUD can extend beyond bedside values, such as respect for patient autonomy and protection of employee safety, to population-level ethical values, such as equitable enforcement of hospital behavioral expectations and the promotion of health equity via wise allocation of hospital resources. Healthcare workers should protect the therapeutic environment of the inpatient unit by prohibiting violence and any manifestations of SUD that truly endanger other patients without forcing PWUD to comply with potentially biased perceptions of what a hospital unit or healing should look like.

Bias mitigation is critical throughout management of challenging behaviors in PWUD. Healthcare workers may focus on the unacceptability of challenging behaviors in the therapeutic environment due to fears of personal endangerment or disruption of duties. PWUD, by contrast, can experience opioid withdrawal, inadequate pain treatment, and stigma as well as discrimination from healthcare workers [11–13], fueling a desire to depart the hospital unit, use drugs in the hospital, or otherwise exhibit challenging behaviors [14].

Identifying these ethical conflicts is the first step toward preventing escalating challenging behaviors or responding when prevention fails.

## THE PREVENTION OF CHALLENGING BEHAVIORS IN HOSPITALIZED PATIENTS

All hospitalized patients should receive nonstigmatizing, patient-centered care. Sadly, stigmatizing language and discrimination toward PWUD are common and, we believe, contribute to the emergence of challenging behaviors in hospitalized PWUD [15, 16].

Patient and clinician contributions to challenging behaviors in hospitalized PWUD can be mitigated via the provision of patient-centered care informed by harm reduction and addiction treatment expertise. Patient-centered care involves forming a therapeutic alliance via nonjudgmental and empathic interactions, practicing trauma-informed care, providing culturally safe care [17], and practicing shared decision-making [18]. Inpatient clinicians who educate patients about wound care, safer injection, and safer sex practices can build therapeutic alliances with respectful sharing of expertise that can support patient engagement in care for PWUD [19–21]. Coupled with de-escalation training, these approaches can help clinicians avoid unnecessary conflicts with PWUD [22].

Harm reduction should be woven into routine care for PWUD. Harm reduction is a set of practical strategies aimed at reducing the negative consequences of drug use while respecting the rights of PWUD [23, 24]. In the hospital, harm reduction–based care involves transparently discussing safer drug use, screening for infections, administering vaccines, prescribing pre- and post-exposure prophylaxis for human immunodeficiency virus and other sexually transmitted infections, providing safer use equipment, referring individuals to syringe services programs, and providing naloxone upon hospital discharge [25]. Some hospitals outside of the United States support safe injection in the hospital to reduce the risk of fatal overdose and enhance connections to other forms of needed care [26]. Harm reduction–based care also involves undertaking nonjudgmental shared decision-making about clinical decisions, for example, choosing intravenous versus oral antibiotics or operative versus nonoperative management of valvular infection [27]. Harm reduction approaches that value patient autonomy can also minimize undesirable outcomes such as patient-directed discharges [28].

Addiction consultation services are also the standard of care for PWUD. MOUD, with wrap-around services provided by addiction medicine experts, is associated with treatment uptake for SUD, reduction of hospital readmissions, and development of a therapeutic alliance, particularly when a peer support worker is integrated into the team [29, 30].

Overly restrictive and inflexibly applied hospital policies, such as mandatory searches of patients’ belongings or prohibiting substance use, can lead to stigma or even discrimination toward PWUD. Negative patient–clinician interactions such as these can fuel needless conflict between patients and clinicians and, by extension, poor outcomes from patient-directed discharges to deaths. Ideally, policies should prioritize both the health of the patient and their care teams [31, 32] via plans created by nonjudgmental conversations between patients and care teams that have sufficient training in the assessment and treatment of pain, withdrawal symptoms, and SUD [33]. This may require multidisciplinary revision of existing policies with input from addiction consultation services to ensure policies are aligned with best practices, equitable, and nonstigmatizing [31, 34].

In-hospital drug use can be a divisive topic. Some clinicians believe in-hospital drug use is an intolerable behavioral violation and risky to patients and clinicians. Others believe drug use is an unavoidable and unsurprising manifestation of SUD, particularly when patients are in pain or withdrawal. If in-hospital drug use occurs, it can be profitable to engage in care team education on harm reduction approaches and de-escalation techniques, such as the use of verbal and nonverbal communication to minimize aggression [35]. We encourage care teams to focus not on drug use itself but rather on measures to protect the safety of patients (such as from overdoses) and employees (such as from needlesticks). A collaborative team approach can mitigate the need for the involvement of hospital security [20, 34], especially given frequent PWUD anecdotes of negative experiences with the carceral setting and stigmatizing interactions with hospital security.

Beyond minimizing negative patient experiences, staff appreciate the provision of care team education around harm reduction, trauma-informed care, and patient safety plans [20, 33].

## WHEN PREVENTION FAILS: URGENCY-APPROPRIATE MANAGEMENT OF CHALLENGING BEHAVIORS

Medical urgency and risks associated with challenging behaviors rightly influence clinicians’ management of challenging behaviors in hospitalized PWUD, as summarized in Table 1.

**Table 1.** Classifications of Challenging Behaviors in Hospitalized People Who Use Drugs Based on Patient Stability and Behavioral Risk

Classification		Example Patient Clinical Scenario	Example Responses^c^
Patient Clinical Status^a^	Safety Risk^b^ of Patient Behavior		
Stable	Low	Drained leg abscess that requires wound care; patient would like to go outside to smoke	If patient declines alternate nicotine delivery method, agree on time-limited smoke breaks with staff notification in a fashion that does not interfere with indicated diagnostics or treatments
Stable	High	Foot ulcer and osteomyelitis that required surgical debridement; patient threatening to harm staff if pain control is not optimized	Assess for undertreated infection and pain as well as any psychiatric contributors to patient behaviors in a nonstigmatizing fashion; clarify behavioral expectations and consequences of nonadherence; if threatening behavior persists despite addressing pain control, discharge the patient with antibiotics, naloxone, and follow-up care for preventive and treatment services
Unstable	Low	Vertebral osteomyelitis with cord compression, awaiting urgent neurosurgical decompression in the operating room; patient is wandering the unit and other floors to relieve their anxiety as they withdraw	Ensure sufficient pain control, for example, via short-acting opioids in addition to adequate use of medications for opioid use disorder; offer any appropriate treatments for anxiety; reassure patient surgery will happen soon; encourage patient to stay near room so transport finds them when they arrive
Unstable	High	Infective endocarditis with refractory heart failure in need of urgent valve repair; patient is highly intoxicated and tries to punch bedside nurse	Sedate patient to enable life-saving surgery and protect staff safety; reevaluate behavioral management plan once postoperative sedation is lightened

^a^“Stable” connotes negligible risk of death or serious injury if treatment is discontinued or transitioned to outpatient care; “unstable” indicates the opposite.

^b^Safety risk could pertain to risk to the patient, other patients, or employees.

^c^All examples assume the failure of routine preventive measures including compassionate clinician communications.

When stable PWUD exhibit nonviolent, challenging behaviors, clinicians can negotiate reasonable behavioral expectations and make accommodations. In such cases, clinicians should examine how the situation affects canonical bioethical values such as respect for autonomy and justice while also examining the microethics of routine clinical interactions, from the way we describe patients with colloquial or nonstigmatizing labels to how clinicians can empower patients in clinical decision-making [36–38]. This approach requires clinicians to examine their own biases and how those biases or prior adverse treatment in healthcare settings may contribute to undesired patient behaviors, as well as judgments about whether challenging patient behaviors are truly dangerous versus irksome. This is also an opportunity to involve the collective efforts of a multidisciplinary team, from nurses and social workers to patients, ethicists, and beyond.

When stable PWUD exhibit violence or other high-risk challenging behaviors [39], clinician responses, which are best formulated as a team and with support from experts such as a psychiatrist, typically hinge on patient decision-making capacity. To protect employee safety, clinicians can insist that patients with intact decision-making capacity improve their behavior with support from adequate MOUD and pain control, or lose access to needed care. Violent patients with impaired decision-making capacity may require sedation to prevent harm to themselves or others.

When clinical and behavioral urgency intersect, clinicians must protect the patient's life while taking any necessary steps to ensure clinical care is safe to provide. Examples include initiating involuntary psychiatric holds or the use of physical or chemical restraints to ensure patient survival without compromising clinician and staff safety. Regardless of the intervention, the priority in such scenarios is to intervene in a way that minimizes infringement on the patient’s autonomy and dignity. By making decisions that are aimed at alleviating patient suffering while prioritizing the safety of all involved, clinicians can remain partners rather than adversaries with their patients and colleagues.

## CASE RESOLUTION

The clinician team, including addiction psychiatry, held a multidisciplinary meeting to discuss Mr. F's care. Physicians wanted hospitalization to continue until the bacteremia cleared and to increase MOUD doses to help the patient avoid independent drug use. The patient agreed to use a sharps container if he used drugs. The team clarified behavioral expectations, including avoidance of threatening or biased language. Mr. F complied. Nursing staff agreed to a predictable schedule of time off the hospital unit that gave Mr. F “the chance to breathe real air” without missing antibiotic doses. Mr. F left the hospital a week later with the bacteremia cleared and followed up at the clinic while on oral antibiotics.

## CONCLUSIONS

Managing challenging behaviors in hospitalized PWUD requires prioritization of ethical values based on clinical urgency, routine investments in harm reduction and conflict de-escalation, and nonpunitive and unbiased team enforcement of behavioral expectations. There are no easy answers, and most cases require team dialogue about ethical tensions to develop a plan to protect both patients and colleagues.

## Note

***Financial support*:** None.
